# Increases in Overdose Deaths Among People With HIV Across 14 U.S. Jurisdictions, 2014–2023

**DOI:** 10.1016/j.focus.2026.100544

**Published:** 2026-08-12

**Authors:** Amy I. Zlot, Steven Erly, Leticia Lawson, Kate Drezner, Cheryl A. Barber, Danica E. Kuncio, O. Ena Omoike, Janae Benning, Alagie Jatta, Joyce Mbugua, Annabel Nicholas, Eleanor Garlow, Riley Gulbronson, Laura Kersanske, Maxwell Short, Randall Collura, Megan Duffy, Melissa M. Kim, Kathleen A. Brady, Jeff Capizzi

**Affiliations:** 1 Oregon Health Authority, Portland, Oregon; 2 Office of Infectious Disease, Washington State Department of Health, Olympia, Washington; 3 Department of Epidemiology, University of Washington, Seattle, Washington; 4 DC Department of Health, Washington, District of Columbia; 5 Minnesota Department of Health, St. Paul, Minnesota; 6 New Jersey Department of Health, Trenton, New Jersey; 7 Michigan Department of Health and Human Services, Lansing, Michigan; 8 Iowa Department of Health and Human Services, Des Moines, Iowa; 9 Division of Epidemiology, Georgia Department of Public Health, Atlanta, Georgia; 10 Tennessee Department of Health, Nashville, Tennessee; 11 Massachusetts Department of Public Health, Boston, Massachusetts; 12 Arizona Department of Health Services, Phoenix, Arizona; 13 New York State Department of Health, Albany, New York; 14 Colorado Department of Public Health and Environment, Denver, Colorado; 15 Division of HIV Health, Philadelphia Department of Public Health, Philadelphia, Pennsylvania

**Keywords:** HIV, overdose mortality, opioids, psychostimulants, multi-jurisdictional

## Abstract

**Introduction:**

Although overall overdose deaths in the U.S. have plateaued in recent years, there is limited information about how recent changes in the drug landscape have affected people with HIV at regional and national levels. The purpose of this analysis was to describe trends in the cause of death among people with HIV, with a focus on drug-related mortality in this population.

**Methods:**

The authors aggregated cause of death data from HIV surveillance systems in 14 jurisdictions across the U.S. to compare regional trends among people with HIV from 2014 to 2023. Causes of death were categorized as fatal overdose, HIV-related disease, or all other causes based on the underlying ICD-10 codes. Demographic characteristics, HIV care indicators, and substance use trends were also evaluated.

**Results:**

Among people with HIV, the number of overdose deaths increased from 225 in 2014 to 993 in 2023, accounting for 15.6% of all deaths, whereas HIV-related deaths declined from 1,904 to 1,312, representing 20.6% of all deaths in 2023. In the Pacific Northwest and on the East Coast, overdose deaths surpassed HIV-related deaths in 2023. The proportion of overdose deaths among people with HIV attributable to opioids increased in nearly all regions.

**Conclusions:**

Fatal overdose was a major contributor to mortality among people with HIV and, in some regions, exceeded HIV-related mortality. These findings underscore the importance of overdose prevention for people with HIV, who may benefit from syndemic approaches that integrate HIV care with mental health services, social support, and harm reduction interventions.

## Highlights

• Overdose deaths among people with HIV quadrupled, 2014–2023.
• By 2023, drug-related deaths rivaled HIV-related deaths in some regions.
• Opioid deaths rose widely; psychostimulant death patterns varied by region.
• People who inject drugs and adults aged 25–44 years were most affected.
• A syndemic approach linking HIV care with overdose prevention may save lives.

## Graphical Abstract

**Figure:**
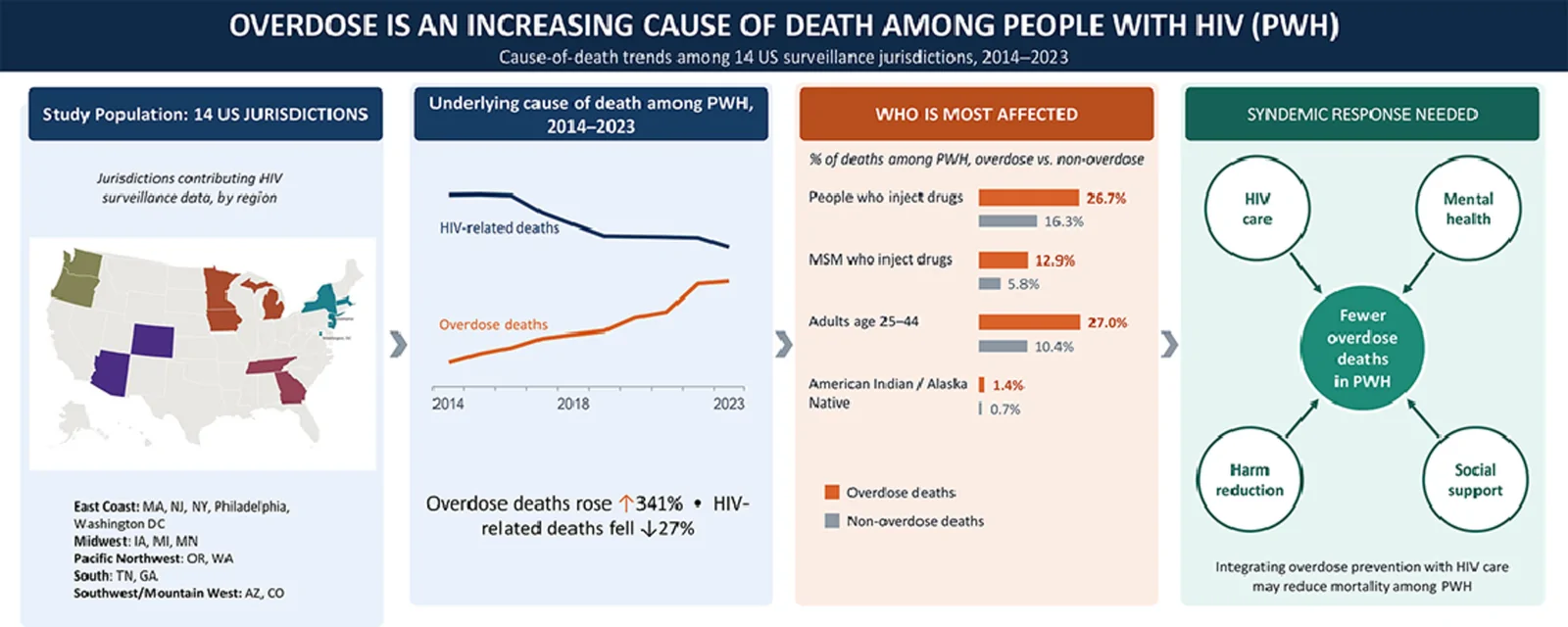

## INTRODUCTION

Drug overdose has emerged as a leading cause of death among people with HIV (PWH), despite declining rates in the general population (a 4% decrease between 2022 and 2023) and a marked expansion in naloxone access, including a more than twofold increase in retail pharmacy dispensing between 2019 and 2023.^1^^,^^2^ Concurrently, PWH are living longer due to antiretroviral therapy and reductions in HIV-related mortality. The risk of overdose may be elevated among PWH, possibly reflecting their disproportionate burden of chronic pain, stigma, and mental health challenges.^3^^,^^4^ In addition, psychostimulant use, such as methamphetamine, is associated with HIV acquisition, higher viral loads, and advanced HIV progression.^5^ Rising psychostimulant-related deaths among PWH reflect both this elevated risk and shifts in the drug supply, including polysubstance use involving fentanyl.^6^

Existing analyses of overdose mortality among PWH, largely limited to single jurisdictions or short time periods, restrict the understanding of regional differences and long-term trends. Although overdose mortality in the U.S. general population rose from 8.9 per 100,000 in 2003 to 32.6 in 2022 before declining to 31.3 in 2023,^7^ it remains unknown whether similar patterns persist among PWH in the U.S., whether overdose deaths have outpaced HIV-related mortality, or whether trends vary by region.^8^, ^9^, ^10^ This study combines underlying cause of death data from 14 jurisdictions across 5 regions to describe trends, characteristics, and substances associated with overdose deaths among PWH from 2014 to 2023.

## METHODS

Aggregated data tables on underlying cause of death for PWH from 2014 to 2023 were provided by 14 Centers for Disease Control and Prevention–funded jurisdictions across 5 regions: Massachusetts, New Jersey, New York, Philadelphia, and Washington, DC (East Coast); Iowa, Michigan, and Minnesota (Midwest); Oregon and Washington (Pacific Northwest); Tennessee and Georgia (South); and Arizona and Colorado (Southwest/Mountain West). Jurisdictions routinely match their HIV surveillance data with death data aggregated from state vital records offices, the National Death Index, and the Social Security Death Index. Matched data are stored in jurisdiction-maintained registries that also contain demographic, clinical, HIV transmission category, and vital status information, as outlined in the Centers for Disease Control and Prevention guidance.^11^ Each jurisdiction tallied all deaths among PWH and categorized them on the basis of the underlying cause of death as (1) overdose (ICD-10 codes X41-X44, X61-X64, or Y11-Y14), (2) HIV-related (ICD-10 codes B20-24, B59, or R75), and (3) all other causes.

For 2023, the authors compared overdose with nonoverdose causes of death (HIV-related and all other causes) by patient sex, race/ethnicity, transmission category, and HIV care status using chi-square tests. Viral suppression was defined as a viral load ≤200 copies/mL within 12 months of death. Not virally suppressed status included a viral load >200 copies/mL or missing data. Characteristics of PWH who were alive as of December 31, 2023 were also described.

Next, trends in overdose and HIV‑related deaths among PWH across the 5 regions from 2014 to 2023 were examined using Poisson regression with a log link (PROC GENMOD, SAS Enterprise Guide 8.6). Annual counts of overdose and HIV‑related deaths were modeled as a function of year, region, and a year-by-region interaction term. Interaction terms were used to assess whether temporal trends differed by region.

The authors further categorized overdose deaths into those involving opioids and psychostimulants only using the underlying and contributing cause of death fields. Opioid-involved overdose deaths were defined as deaths with an underlying overdose cause (ICD-10 × 41–X44, X61–X64, or Y11–Y14) and an opioid poisoning code (ICD-10 T40.1–T40.4) listed anywhere on the death certificate. Psychostimulant-only overdose deaths were defined as deaths with an underlying cause of overdose and a psychostimulant poisoning code (ICD-10 T43.6) on the death certificate, with no opioid poisoning codes listed. This definition included methamphetamine but excluded cocaine because cocaine-related deaths were relatively uncommon in this study population, present in approximately 5% of overdoses. To avoid double counting and because opioids carry a higher acute overdose risk, deaths with both opioid and psychostimulant codes were classified as opioid related. On the basis of data from the Pacific Northwest, where these data are available, approximately 40% to 50% of overdose deaths with a psychostimulant ICD-10 code also included an opioid code as a contributing cause of death.

For small cell sizes, data were suppressed according to jurisdiction-specific guidelines. When aggregating across sites, midpoints were imputed for reported ranges (e.g., <10 was counted as 5).^12^ As a result, categorical totals may not sum to 100%. This study was exempt from IRB review because it involved the analysis of aggregated, deidentified surveillance data.

## RESULTS

In 2023, 6,370 PWH died across the 14 jurisdictions; 993 (15.6%) had a fatal overdose as the underlying cause of death, and 1,312 (20.6%) died from HIV-related causes. Compared with PWH who died from nonoverdose causes, deaths due to overdose were overrepresented among American Indian/Alaska Native people (1.5% vs 0.8%), people who injected drugs (27.6% vs 15.9%), men who have sexual contact with men and also inject drugs (12.9% vs 5.8%), and adults aged 25–44 years (27.9% vs 13.1%) (Table 1).

**Table 1:** Characteristics of PWH Who Died in 2023, by Underlying Cause of Death (*n*=6,370), 2023^a^

Variable	Value	Overdose^b^	HIV^c^	All other causes	Total nonoverdose causes^d^	Total PWH deaths	*p*-value^e^
All	*n* (% of total PWH deaths)	993 (15.6%)	1,312 (20.6%)	4,065 (63.8%)	5,377 (84.4%)	6,370 (100.0%)	
Sex at birth, *n* (column %)	Male	710 (71.5%)	985 (75.1%)	2,821 (69.4%)	3,806 (70.8%)	4,516 (70.9%)	0.3322
	Female	247 (24.9%)	324 (24.7%)	1,107 (27.2%)	1,431 (26.6%)	1,678 (26.3%)	
	Missing	36 (3.6%)	3 (0.2%)	137 (3.4%)	140 (2.6%)	176 (2.8%)	
Race/ethnicity, *n* (column %)	American Indian/Alaska Native	14 (1.4%)	15 (1.1%)	27 (0.7%)	42 (0.8%)	56 (0.9%)	00.0485
	Black/African American	437 (44.0%)	619 (47.2%)	1,767 (43.5%)	2,386 (44.4%)	2,823 (44.3%)	
	Hispanic/Latine	180 (18.1%)	247 (18.8%)	674 (16.6%)	921 (17.1%)	1,101 (17.3%)	
	White	266 (26.8%)	348 (26.5%)	1,309 (32.2%)	1,657 (30.8%)	1,923 (30.2%)	
	Another race	67 (6.7%)	80 (6.1%)	238 (5.9%)	318 (5.9%)	385 (6.0%)	
	Missing	29 (2.9%)	3 (0.2%)	50 (1.2%)	53 (1.0%)	82 (1.3%)	
Transmission risk, *n* (column %)	Male-to-male sexual contact	271 (27.3%)	463 (35.3%)	1,333 (32.8%)	1,796 (33.4%)	2,067 (32.4%)	<0.0001
	IDU	265 (26.7%)	195 (14.9%)	662 (16.3%)	857 (15.9%)	1,122 (17.6%)	
	MSM/IDU	128 (12.9%)	69 (5.3%)	236 (5.8%)	305 (5.7%)	433 (6.8%)	
	Heterosexual	145 (14.6%)	236 (18.0%)	708 (17.4%)	944 (17.6%)	1,089 (17.1%)	
	Another transmission risk (nonmissing)	142 (14.3%)	319 (24.3%)	1,126 (27.7%)	1,445 (26.9%)	1,587 (24.9%)	
	Missing	42 (4.2%)	30 (2.3%)	0 (0.0%)	30 (0.6%)	72 (1.1%)	
Age in 2023, *n* (column %)	<25 years	10 (1.0%)	8 (0.6%)	29 (0.7%)	37 (0.7%)	47 (0.7%)	<0.0001
	25–44 years	268 (27.0%)	284 (21.6%)	423 (10.4%)	707 (13.1%)	975 (15.3%)	
	45–64 years	517 (52.1%)	602 (45.9%)	1,681 (41.4%)	2,283 (42.5%)	2,800 (44.0%)	
	≥65 years	167 (16.8%)	412 (31.4%)	1,912 (47.0%)	2,324 (43.2%)	2,491 (39.1%)	
	Missing	31 (3.1%)	6 (0.5%)	20 (0.5%)	26 (0.5%)	57 (0.9%)	
Care status,^f^*n* (column %)	Virally suppressed	534 (53.8%)	737 (56.2%)		2,990 (55.6%)	3,524 (55.3%)	0.9653
	Not virally suppressed	425 (42.8%)	575 (43.8%)		2,387 (44.4%)	2,812 (44.1%)	
	Missing	34 (3.4%)	0 (0.0%)		0 (0.0%)	34 (0.5%)	

a Totals may not sum to 100 percent of the overall count due to missing or incomplete data and because midpoints were used when data for individual jurisdictions were suppressed

b ICD-10 codes X41–X44, X61–X64, or Y11–Y14.

c ICD-10 codes B20–24, B59, or R75.

d Includes HIV and other causes of death.

e Chi-square test: Overdose deaths compared with all nonoverdose causes of death (HIV + another cause).

f Virally suppressed defined as having a viral load test ≤200 copies/mL within 12 months of death. Not virally suppressed category includes individuals missing viral load information. IDU, injection drug use; MSM, men sex men; PWH, people with HIV.

Between 2014 and 2023, the number of overdose deaths increased across all jurisdictions, whereas the number of HIV-related deaths declined in every region except the Midwest and the Pacific Northwest. In Poisson regression models, overdose deaths rose by an average of 16% per year overall, with significant annual increases in every region (*p*<0.0001), ranging from approximately 15% per year on the East Coast to 26% per year in the Pacific Northwest and a 21% per-year increase in the South. Over the same period, HIV-related deaths decreased by 5% per year overall, with significant declines in most regions but little to no change in the Midwest and the Pacific Northwest. By 2023, overdose deaths exceeded HIV-related deaths in the Pacific Northwest and East Coast and approached similar levels in the Southwest/Mountain West (Figure 1).

**Figure 1:**
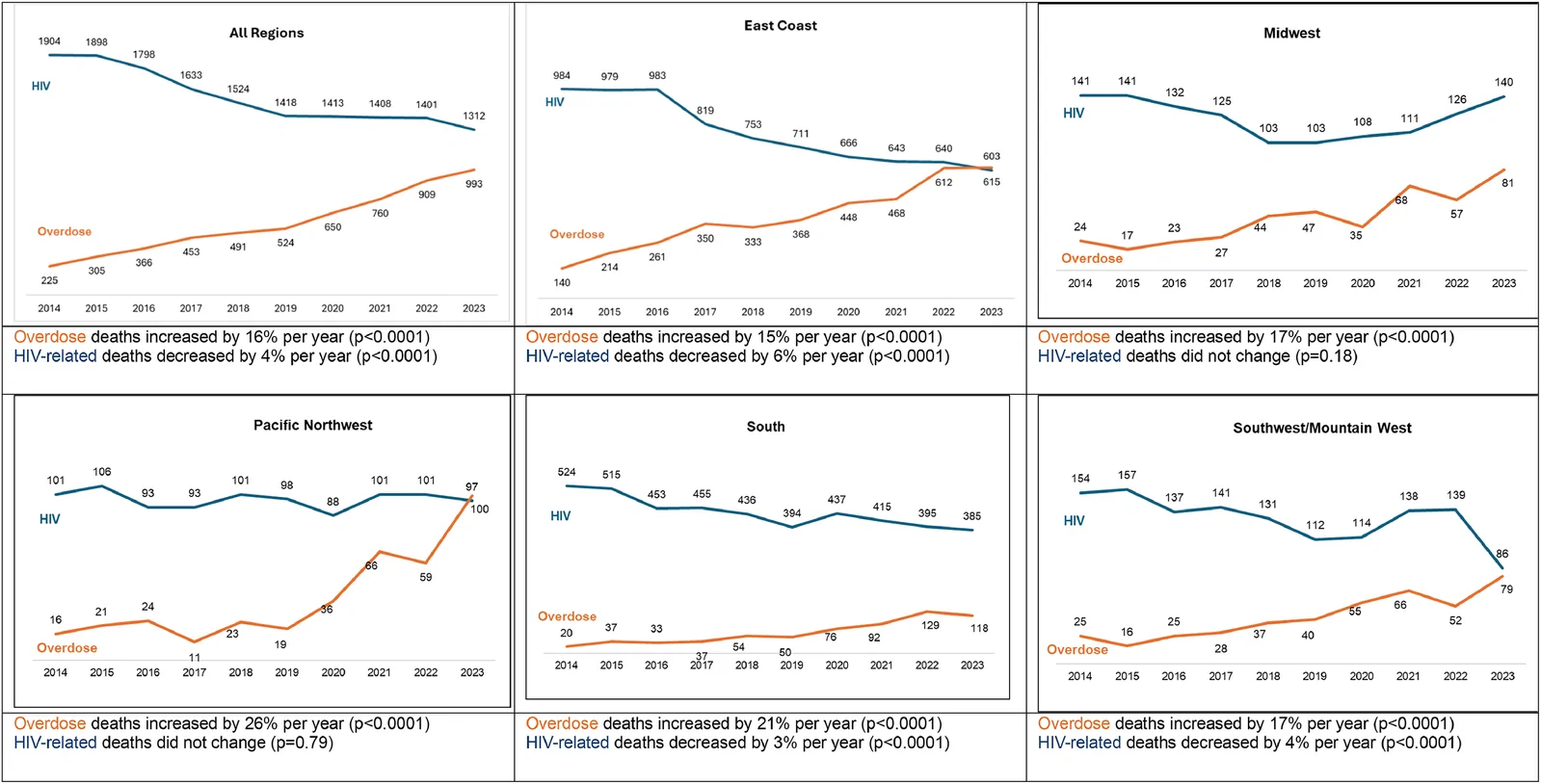
Number of HIV and overdose deaths as underlying causes of death among PWH by region and year (2014–2023). *Note*: Regions are defined as follows: East Coast: Massachusetts, New Jersey, New York, Philadelphia, and Washington, DC; Midwest: Iowa, Michigan, and Minnesota; Pacific Northwest: Oregon and Washington; South: Tennessee and Georgia; and Southwest/Mountain West: Arizona, Colorado. Deaths were identified from pooled HIV surveillance data from state and city jurisdictions. HIV as an underlying cause of death was defined using ICD-10 codes B20–B24, B59, or R75; fatal overdose as an underlying cause was defined using ICD-10 codes X41–X44, X61–X64, or Y11–Y14. Y-axis scales vary across the regional trend graphs to improve the visibility of data points. Poisson regression models were used to compare trends in overdose and HIV-related deaths among PWH across 5 regions from 2014 to 2023 using Poisson regression with a log link (PROC GENMOD, SAS Enterprise Guide 8.6). PWH, people with HIV.

From 2016 to 2023, the absolute number of opioid- and psychostimulant-attributable overdose deaths increased, with opioid-attributable deaths rising by 23% per year overall (*p*<0.0001) and psychostimulant-only deaths increasing by 17% per year. Over the same period, the proportions of overdoses involving these substances fluctuated, with distinct regional patterns. Opioid-attributable overdoses increased in all regions except the Southwest/Mountain West, where the proportions were similar in 2016 and 2023 (48.0% vs 48.9%). On the East Coast, overdoses attributed only to psychostimulants never exceeded 10%. In the Southwest/Mountain West, psychostimulant-attributed overdoses declined from 19.3% in 2016 to 9.6% in 2023. In the Pacific Northwest, psychostimulant-attributable overdoses peaked at 52.6% in 2019 and declined to 26.0% by 2023. In the South, the proportion of overdoses attributed to psychostimulants doubled between 2016 and 2017, peaking at 32.4% in 2017 before declining to an average of 20% from 2021 to 2023 (Figure 2).

**Figure 2:**
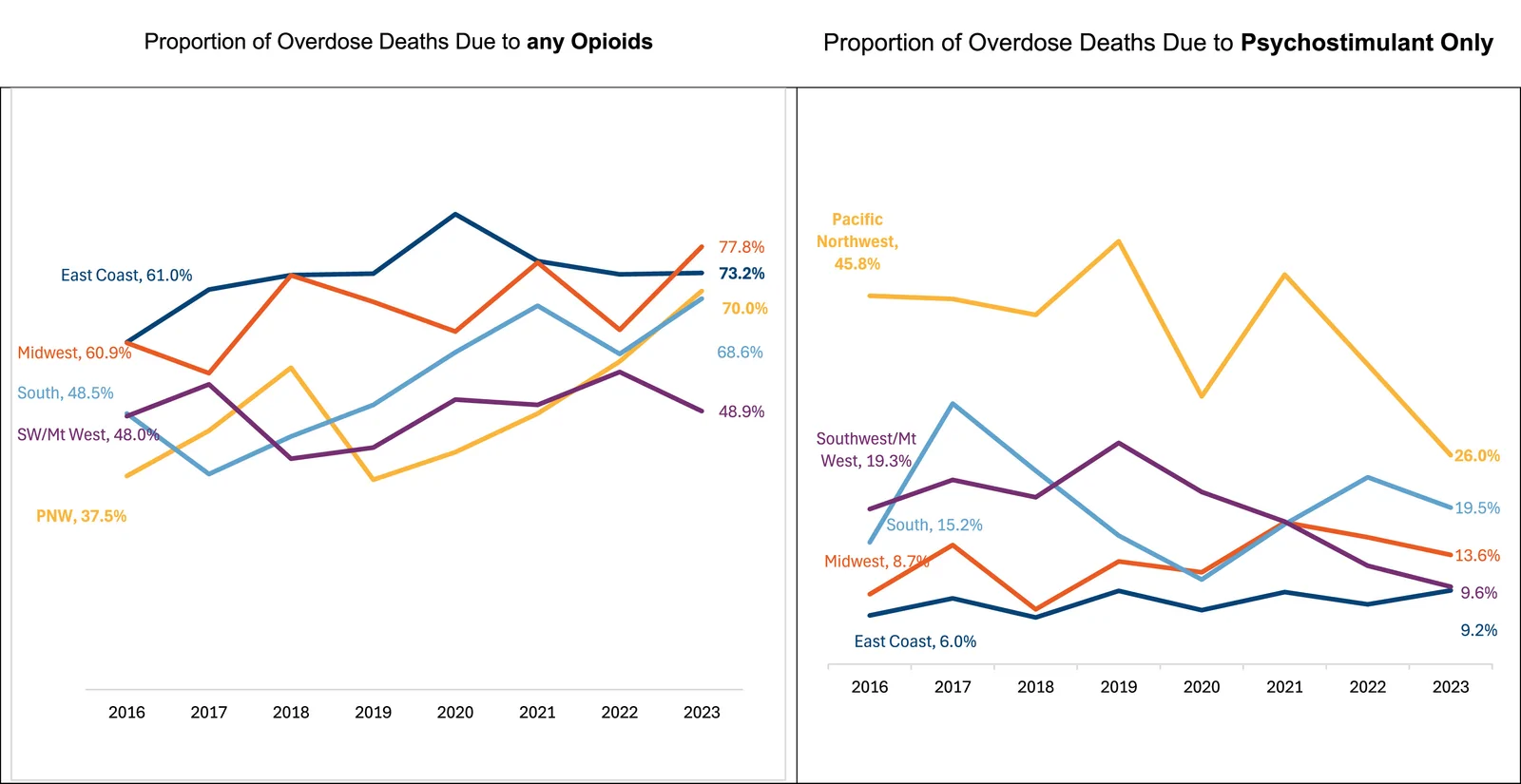
Proportion of overdose deaths among people with HIV attributed to any opioids and to psychostimulants only, by jurisdiction, 2016–2023. *Note*: Regions are defined as follows: East Coast: Massachusetts, New Jersey, New York, Philadelphia, and Washington DC; Midwest: Iowa, Michigan, and Minnesota; Pacific Northwest: Oregon and Washington; South: Tennessee and Georgia; Southwest/Mountain West: Arizona and Colorado. Deaths were identified from pooled HIV surveillance data from state and city jurisdictions. Substance involvement was categorized as follows: any opioids: underlying cause of death overdose (ICD-10 codes X41–X44, X61–X64, or Y11–Y14) with at least 1 contributing cause-of-death code (T40.1–T40.4) on the death certificate; psychostimulants only: underlying cause of death overdose (ICD-10 codes X41–X44, X61–X64, or Y11–Y14) with contributing cause-of-death code T43.6 on the death certificate and no T40.1–T40.4 codes present.

## DISCUSSION

People with HIV are disproportionately affected by overdose deaths,^8^^,^^13^ which increased more than fourfold across the 14 jurisdictions from 2014 to 2023. Research suggests that addressing substance use among PWH requires syndemic, trauma-informed approaches that strengthen clinical outcomes, expand mental health and social supports, address socioeconomic disparities, and promote HIV prevention and harm reduction.^14^, ^15^, ^16^

Recently, several U.S. jurisdictions have declared HIV outbreaks among people who inject drugs, reflecting intersecting drivers of injection drug use, unstable housing, and limited access to prevention and harm reduction services. These outbreaks, often involving people experiencing homelessness or living in encampments, underscore the need to expand harm reduction, syringe services, and comprehensive care for PWH.^17^^,^^18^ This includes long-acting injectable antiretroviral therapy, which provides additional options to support HIV treatment and prevention.

Harm reduction approaches, including syringe service programs, not only reduce HIV transmission but are also crucial for linkage to substance use treatment and for the distribution of fentanyl test strips and naloxone. Although polysubstance use was not directly examined in this study, recent analyses indicate that the opioid epidemic has evolved into a dual-substance epidemic involving both fentanyl and stimulants.^19^^,^^20^

Interventions for opioid and methamphetamine use differ substantially. Naloxone effectively reverses opioid overdose, but no pharmaceutical exists to reverse psychostimulant overamping, and there is no Food and Drug Administration–approved medication for stimulant use disorder.^21^ Nonpharmaceutical approaches, such as contingency management, particularly when peer supported, have demonstrated effectiveness and can be lifesaving.^22^

### Limitations

There were several limitations to this analysis. First, overdose deaths were not classified by intent, combining unintentional and intentional overdoses. Second, the available racial categories were limited and may obscure differences within broader groups. Third, all comparisons were conducted without adjustment for confounding factors, particularly age distribution. Fourth, the 14 jurisdictions included represent a convenience sample of those that agreed to participate and are not fully representative of all regions; however, within each participating jurisdiction, all diagnosed HIV cases that died within the study period were included. Finally, the analysis does not account for polysubstance use, which may influence overdose patterns and intervention needs.

## CONCLUSIONS

Overdose-related deaths may contribute to reduced life expectancy among PWH. Low-barrier, stigma-free, coordinated HIV prevention, substance use disorder, and harm reduction interventions, along with structural supports that address social determinants of health, are essential to mitigating premature mortality.

## CRediT authorship contribution statement

**Amy I. Zlot:** Conceptualization, Data curation, Writing – original draft. **Steven Erly:** Data curation, Formal analysis, Writing – review & editing. **Leticia Lawson:** Data curation, Formal analysis, Writing – review & editing. **Kate Drezner:** Data curation, Formal analysis, Writing – review & editing. **Cheryl A. Barber:** Data curation, Formal analysis, Writing – review & editing. **Danica E. Kuncio:** Data curation, Formal analysis, Writing – review & editing. **O. Ena Omoike:** Data curation, Formal analysis, Writing – review & editing. **Janae Benning:** Data curation, Formal analysis, Writing – review & editing. **Alagie Jatta:** Data curation, Formal analysis, Writing – review & editing. **Joyce Mbugua:** Data curation, Formal analysis, Writing – review & editing. **Annabel Nicholas:** Data curation, Formal analysis, Writing – review & editing. **Eleanor Garlow:** Data curation, Formal analysis, Writing – review & editing. **Riley Gulbronson:** Data curation, Formal analysis, Writing – review & editing. **Laura Kersanske:** Data curation, Formal analysis, Writing – review & editing. **Maxwell Short:** Data curation, Formal analysis, Writing – review & editing. **Randall Collura:** Data curation, Formal analysis, Writing – review & editing. **Megan Duffy:** Data curation, Formal analysis, Writing – review & editing. **Melissa M. Kim:** Data curation, Formal analysis, Writing – review & editing. **Kathleen A. Brady:** Data curation, Formal analysis, Writing – review & editing. **Jeff Capizzi:** Conceptualization, Formal analysis, Writing – review & editing.
